# Case series: Cervical far-lateral and combined cervical far lateral/foraminal intervertebral disk extrusions in 10 dogs

**DOI:** 10.3389/fvets.2024.1465182

**Published:** 2024-11-06

**Authors:** Koen M. Santifort, Sergio Gomes, Marco Ruggeri, Emili Alcoverro, Anna Tauro, Esther Lichtenauer, Iris Van Soens, Laurent Garosi, Ines Carrera, Niklas Bergknut, Alba Farre Marine, Alejandro Luján Feliu-Pascual

**Affiliations:** ^1^IVC Evidensia Small Animal Referral Hospital Arnhem, Arnhem, Netherlands; ^2^IVC Evidensia Small Animal Referral Hospital Hart van Brabant, Waalwijk, Netherlands; ^3^Dovecote Veterinary Hospital, Castle Donington, United Kingdom; ^4^ChesterGates Veterinary Specialists, Chester, United Kingdom; ^5^Department of Clinical Sciences, North Carolina State University, Raleigh, NC, United States; ^6^AniCura Ars Veterinària Hospital Veterinari, Barcelona, Spain; ^7^Vet Oracle Teleradiology, Norfolk, United Kingdom; ^8^Aúna Especialidades Veterinarias IVC Evidensia, Paterna, Spain

**Keywords:** herniation, hyperesthesia, chondrodystrophic, pain, nerve root signature

## Abstract

Far-lateral intervertebral disk extrusions (IVDEs) have been reported infrequently in dogs in veterinary literature, mostly affecting the caudal lumbar intervertebral disks. We describe the clinical findings, computed tomography (CT) and magnetic resonance imaging (MRI) findings, treatment, and outcome in 10 dogs with cervical far-lateral IVDEs. Patient databases of 3 small animal hospitals and 1 veterinary teleradiology service were retrospectively searched for patients in which imaging studies (CT or MRI) identified the presence of intervertebral disk material outside the limits of the intervertebral foramen. Presenting clinical signs included: episodic signs of cervical pain (6/10, 30%), persistent signs of cervical pain (3/10, 50%), nerve root signature or lameness (5/10, 50%), and abnormal cervical posture only (excluding nerve root signature) (1/10, 10%). Affected IVD spaces (for 11 IVDEs in 10 dogs) included: C3-4 (6/11, 55%), C5-6 (3/11, 27%), and C2-3 (2/11, 18%). Nerve root signature was not reported for C2-3 IVDEs. All cases were managed medically (without surgery). The top 3 used medications were gabapentinoids (10/10, 100%), non-steroidal anti-inflammatory drugs (NSAIDs) (10/10, 100%), and paracetamol (3/10, 30%). Median treatment duration was 25 days (range 10–84). Short-term outcome (<3 months) was recorded in 9/10 (90%) cases. Resolution of clinical signs was reported in 7/9 (78%) cases. Long-term follow-up was available for 6/10 (60%) cases (median 11.5 months, range 5.5–30 months); 5/6 (83%) showed resolution of clinical signs. Recurrence of clinical signs was reported in 1 case (9 months later), managed medically again, with successful outcome. In conclusion, cervical far-lateral disk extrusions are a rare clinical entity in dogs, but can result in severe, persistent or episodic, pain. Medical management is associated with a positive short- and long-term outcome in most cases.

## Introduction

**Figure 1:**
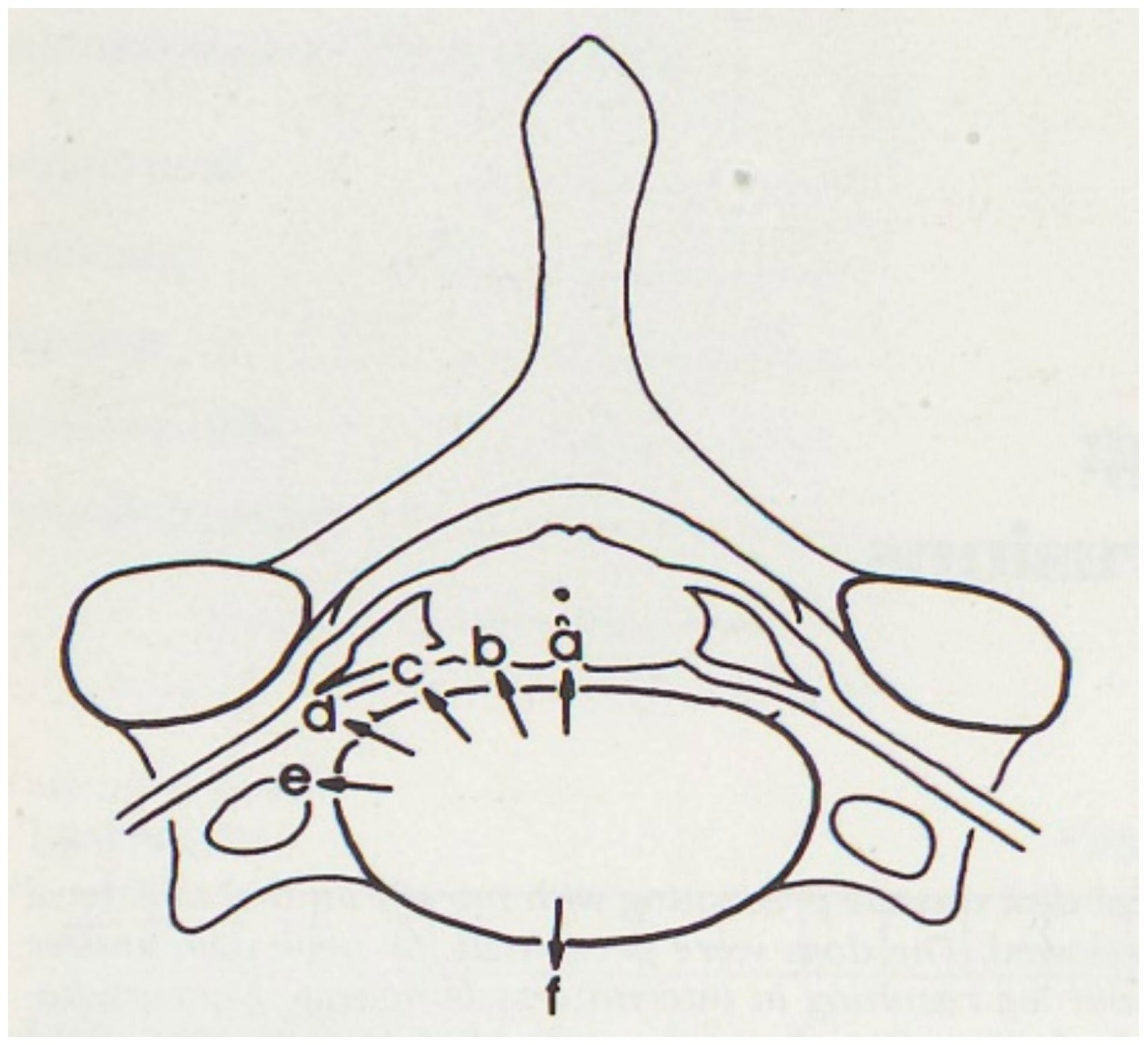
Possible directions of intervertebral disk extrusions. a: dorsomedian, b: paramedian, c: dorsolateral, d: (intra)foraminal, e: far-lateral, f: ventral. Reprinted with permission and legend adjusted from (13).

Intervertebral disk disease is the most common spinal disorder diagnosed in dogs (1–5). A recently published classification scheme describes multiple variants of canine intervertebral disk herniation: intervertebral disk protrusion (IVDP), intervertebral disk extrusion (IVDE), IVDE with extensive epidural hemorrhage (DEEH), traumatic IVDE, intradural/intramedullary IVDE, acute non-compressive nucleus pulposus extrusion (ANNPE) and hydrated nucleus pulposus extrusion (HNPE) (2). This scheme can be complemented with intradural/extramedullary IVDE and lateral IVDE. The latter can be subdivided as lateral IVDE within the spinal canal (i.e., lateralized or dorsal paramedian), foraminal IVDE, and far-lateral IVDE (Figure 1) (6–11). Moreover, one single herniation may be best represented by a combination of two types (e.g., foraminal and far-lateral IVDE) and a single patient may have multiple herniations of the same or different types (7).

Cervical foraminal IVDEs have been reported to occur in dogs, defined as ‘*the presence of intervertebral disk material within the foraminal space in the cervicothoracic spine not associated with spinal cord compression*’ (12). In general terms, the neurovascular foramen may be defined as the opening between adjacent vertebrae through which the spinal nerve (roots) and vessels exit the spinal canal. Often, the spinal nerve roots will join to form the spinal nerve either just within the spinal canal or in the foramen itself. A specific anatomical description of the neurovascular intervertebral foramina in the cervical region of dogs is not noted in literature. Authors have mentioned the foramina to have an ‘entrance’ and ‘exit’ zone (12). A middle part as for lumbosacral foramina is not included, likely due to the comparatively small dimensions (13). Far-lateral IVDEs have been reported infrequently in dogs in the veterinary literature (7–11). Far-lateral IVDE is defined as ‘*the presence of intervertebral disk material outside the limits of the foramen*’ (7). In all of the reported cases, the extrusions were located in the thoracolumbar area, mostly affecting the caudal lumbar intervertebral disks.

There is only one report from 1983 that documents clinical and radiographic findings as well as surgical treatment and outcome in 2 dogs with cervical far-lateral IVDEs (and 5 dogs with foraminal cervical IVDEs) (14). In this retrospective study, we describe the clinical presentation, computed tomography (CT) and magnetic resonance imaging (MRI) findings, treatment, and outcome in 10 dogs with cervical far-lateral and far-lateral/foraminal IVDEs.

## Materials and methods

Patient databases of 3 small animal hospitals and 1 veterinary teleradiology service were retrospectively searched for patients in which imaging studies (CT or MRI) identified the presence of (suspected) intervertebral disk material outside the limits of the foramen or both outside and within the limits of the foramen (7). CT studies were reconstructed in a bone and soft tissue algorithm and displayed in a bone and soft tissue window width and level, and all included myelograms. MRI studies included at least the following sequences: T2-weighted (T2W) sagittal and T2W transverse. When available, short-tau inversion recovery (STIR) dorsal, T2* gradient echo transverse, T1W post-contrast and T1 fat saturation (Fatsat) post-contrast were included. Exclusion criteria were identification of lateralized extradural lesion within the vertebral canal only or concurrent cervical non-IVD related pathology that may have contributed to the clinical signs.

The following imaging features were assessed:

1. Presence or absence of intervertebral disk degeneration and/or mineralization, and intervertebral disk space width;
2. Location of the extradural lesion;
3. Extension of the extradural lesion (within the intervertebral foramen and/or outside the intervertebral foramen along the vertebral body, ventral to the vertebral body);
4. Signal intensity pattern in MRI or CT. This included: (a) homogeneous/heterogenous, (b) signal intensity in all MRI sequences compared to the spinal cord (isointense, hypointense, hyperintense), (c) attenuation in CT compared to soft tissues (isoattenuating, hypoattenuating, hyperattenuating). The Hounsfield Units (HU) of the hyperattenuating lesions were noted in order to assess if the hyperattenuating lesions were or not mineralized (mineralization >500HU);
5. Visualization of vertebral artery and/or spinal nerve at the level of the extradural lesion;
6. Size and signal intensity or attenuation of the spinal nerve at the level of the extradural lesion;
7. Signal intensity or attenuation or the perineural tissues and epaxial muscles at the level of the extradural lesion.

CT and MR images were reviewed by ECVDI and/or ECVN diplomates at initial presentation, and retrospectively reviewed by an ECVDI diplomate. Surgical or histological confirmation of the nature of the material was not required (therefore ‘suspected’). Patient signalment factors (breed, sex and neuter status, age, body weight), clinical history (duration and presenting clinical signs), clinical and neurological examination findings, treatment, and outcome (short-term (< 3 months) and, when available, long-term (>3 months)) were recorded from patient records (i.e., veterinary clinical evaluation by referral clinician or referring veterinarian) and/or telephone consultation at the time of writing. Dogs were considered large breed dogs when body weight was >25 kg, medium when weight was 10–25 kg and small when <10 kg. Descriptive statistics are reported.

## Results

Ten (10) cases met the inclusion criteria (Table 1). Six (6) were male (3 neutered, 3 entire) and 4 were female (2 neutered, 2 entire). Breeds included 2 large breed dogs, 4 medium breed dogs and 4 small breed dogs. Specifically, the following breeds were included: 2 Dachshunds, 2 crossbreeds (chondrodystrophic phenotype), 1 English Cocker Spaniel, 1 French bulldog, 1 Australian kelpie, 1 Beagle, 1 Dalmatian, and 1 Pit bull terrier. Median age was 7 years and 6 months (range 4 years and 2 months to 9 years and 8 months). Median weight was 14.3 kg (range 5.0–29.0).

**Table 1:** Details of the included cases.

Case number Breed Sex and neuter status Age (years, months) Body weight (kg)	History and duration of signs	Clinical findings	Imaging findings	Treatment	Outcome
#1 Pit bull terrier ME 8y6m 28.5 kg	Acute-onset cervical pain after traumatic incident – 2 weeks	Cervical hyperesthesia with left-lateralized on palpation	CT myelogram – left C3-4 far-lateral and foraminal IVDE	Paracetamol (10 mg/kg q8h for 10 days), carprofen (2 mg/kg q12h for 7 days), pregabalin (3.5 mg/kg q12h for 15 days), tramadol (2 mg/kg q8h for 7 days)	Resolution of signs within 2 weeks. Last follow-up 5.5 months.
#2 English Cocker Spaniel MN 9y6m 14.2 kg	Acute-onset cervical pain and right thoracic limb lameness – 1 week, progressive	Cervical hyperesthesia, nerve root signature right thoracic limb	CT myelogram – right C3-4 far-lateral and foraminal IVDE	Carprofen (2 mg/kg q12h for 10 days), pregabalin (4.2 mg/kg q12h for 20 days), tramadol (1 mg/kg q12h for 15 days), paracetamol (9.6 mg/kg q12h for 15 days)	No improvement despite treatment. Euthanasia because of an independent neoplasia 1 year after diagnosis.
#3 Beagle FE 8y5m 14.3 kg	Acute-onset intermittent cervical pain, yelping episodes, and left thoracic limb non-weight bearing / nerve root signature – 10 days	Left thoracic nerve root signature / non-weight bearing lameness, cervical hyperesthesia, reduced withdrawal reflex on the left thoracic limb	CT and low-field MRI – left C5-C6 far-lateral IVDE	Cage rest 4 weeks, meloxicam (0.1 mg/kg q24h for 2 weeks), gabapentin (10 mg/kg q8h for 4 weeks)	Lost to follow-up
#4 Australian kelpie MN 9y8m 16.1 kg	Chronic right thoracic limb lameness – acute deterioration with cervical pain 7 days before presentation	Cervical hyperesthesia with right-lateralization on palpation, nerve root signature right thoracic limb	High-field MRI – right C5-6 far-lateral IVDE	Meloxicam (0.1 mg/kg q24h for 2 weeks), gabapentin (10 mg/kg q8h for 6 weeks)	Resolution of signs in <2 weeks. Last follow-up 30 months.
#5 Dalmatian ME 4y2m 29.0 kg	Acute intermittent cervical pain with yelping episodes, left thoracic limb lameness – 2 weeks	Cervical hyperesthesia, intermittent left thoracic limb lameness/nerve root signature	CT and low-field MRI – left C5-C6 far-lateral IVDE	Cage rest 4 weeks, meloxicam (0.1 mg/kg q24h for 2 weeks), gabapentin (10 mg/kg q8h for 4 weeks)	Improvement, with remaining intermittent lameness. Last follow-up 4 weeks.
#6 French bulldog MN 6y 15.5 kg	Acute intermittent cervical pain with yelping episodes – 4 days	Cervical hyperesthesia	High-field MRI – left C2-3 far-lateral IVDE + left C3-4 far-lateral and foraminal IVDE	Pregabalin (1.6 mg/kg q12h for 4 weeks), meloxicam (0.1 mg/kg q24h for 2 weeks), and methocarbamol (20 mg/kg q8h for 4 weeks)	Resolution of clinical signs in <2 weeks. Total follow-up of 4 weeks.
#7 Dachshund FE 7y2m 5.0 kg	Attacks of abnormal cervical posturing – 2 weeks, waning in frequency	None in clinic – video shows fasciculations/jerks of cervical muscles and cervical pseudodystonia with ventroflexion and head tilting/turning (torticollis)	High-field MRI – left C3-4 far-lateral and foraminal IVDE	Meloxicam (0.1 mg/kg q24h for 2 weeks) and gabapentin (10 mg/kg q8h for 2 weeks, followed by q12h for 1 week and then discontinued)	One recurrent attack in first two days of treatment, thereafter no recurrence. Total follow-up of 10 months.
#8 Crossbreed (CD) FN 7y10m 9.3 kg	Acute onset of intermittent cervical pain with yelping episodes and right thoracic limb lameness/nerve root signature – 2 weeks	Cervical hyperesthesia with right-lateralization on palpation, right-sided thoracic limb lameness/nerve root signature	High-field MRI – right C3-4 far-lateral IVDE	Carprofen (2 mg/kg q12h for 2 weeks), gabapentin (11 mg/kg q8h for 3 months), and amitriptyline (1.1 mg/kg q12h) for 3 months. Failure to respond to only carprofen and gabapentin after 3 days. Response after a week of all 3 mentioned medications.	Resolution of signs within 2 weeks. Treatment with gabapentin and amitriptyline was continued for 3 months. Owners reported no recurrence of signs after tapering first gabapentin, thereafter amitriptyline. Recurrence of cervical hyperesthesia 9 months later, managed medically (NSAID, gabapentin) without diagnostic imaging. Resolution of signs within 2 weeks and medication tapered again in 2 months’ time. Total follow-up of 18 months.
#9 Crossbreed (CD) FN 6y6m 6.4 kg	Acute onset intermittent cervical pain with yelping episodes – 4 days	Cervical hyperesthesia	High-field MRI – right C2-3 far-lateral IVDE	Meloxicam (0.1 mg/kg q24h for 2 weeks) and gabapentin (10 mg/kg q8h for 2 weeks)	Owners reported complete resolution of signs 2 weeks later and discontinued medication. Total follow-up of 11 months.
#10 Dachshund ME 6y 9.0 kg	Acute onset intermittent cervical pain with yelping episodes – 2 days	Cervical hyperesthesia with lateralization on palpation	High-field MRI – left C3-4 foraminal and far-lateral disk extrusion	Meloxicam (0.1 mg/kg q24h for 2 weeks) and gabapentin (10 mg/kg q8h for 2 weeks), paracetamol (10 mg/kg q8h for 5 days), methocarbamol (21 mg/kg q8s for 10 days), ketamine (CRI 5ug/kg/m for 24hs), cage rest	Few recurrent attacks the first 2 days. Complete resolution in 5 days. Total follow up 2 weeks (thereafter lost to follow-up)

CD, chondrodystrophic; FE, female entire; FN, female neutered; ME, male entire; MN, male neutered.

Duration of clinical signs before presentation was <1 week in 3/10 (30%) cases and 1–2 weeks in 7/10 (70%) cases. The clinical history included: episodic signs of cervical pain (6/10, 60%), persistent signs of cervical pain (3/10, 30%), nerve root signature or lameness (5/10, 50%), and abnormal cervical posture only (excluding nerve root signature) (1/10, 10%). Clinical signs and neurological examination abnormalities included: cervical hyperesthesia (9/10, 90%) [lateralization noted: 4/10, 40%, no lateralization noted: 6/10, 60%]; nerve root signature or lameness (5/10, 50%); reduced withdrawal reflex of the ipsilateral thoracic limb (1/10, 10%); no clinical signs in the clinic (i.e., only signs recorded on videos) (1/10, 10%) and; muscle fasciculations/jerks in the cervical musculature (1/10, 10%). Nerve root signature was not reported for C2-3 IVDEs.

**Figure 2:**
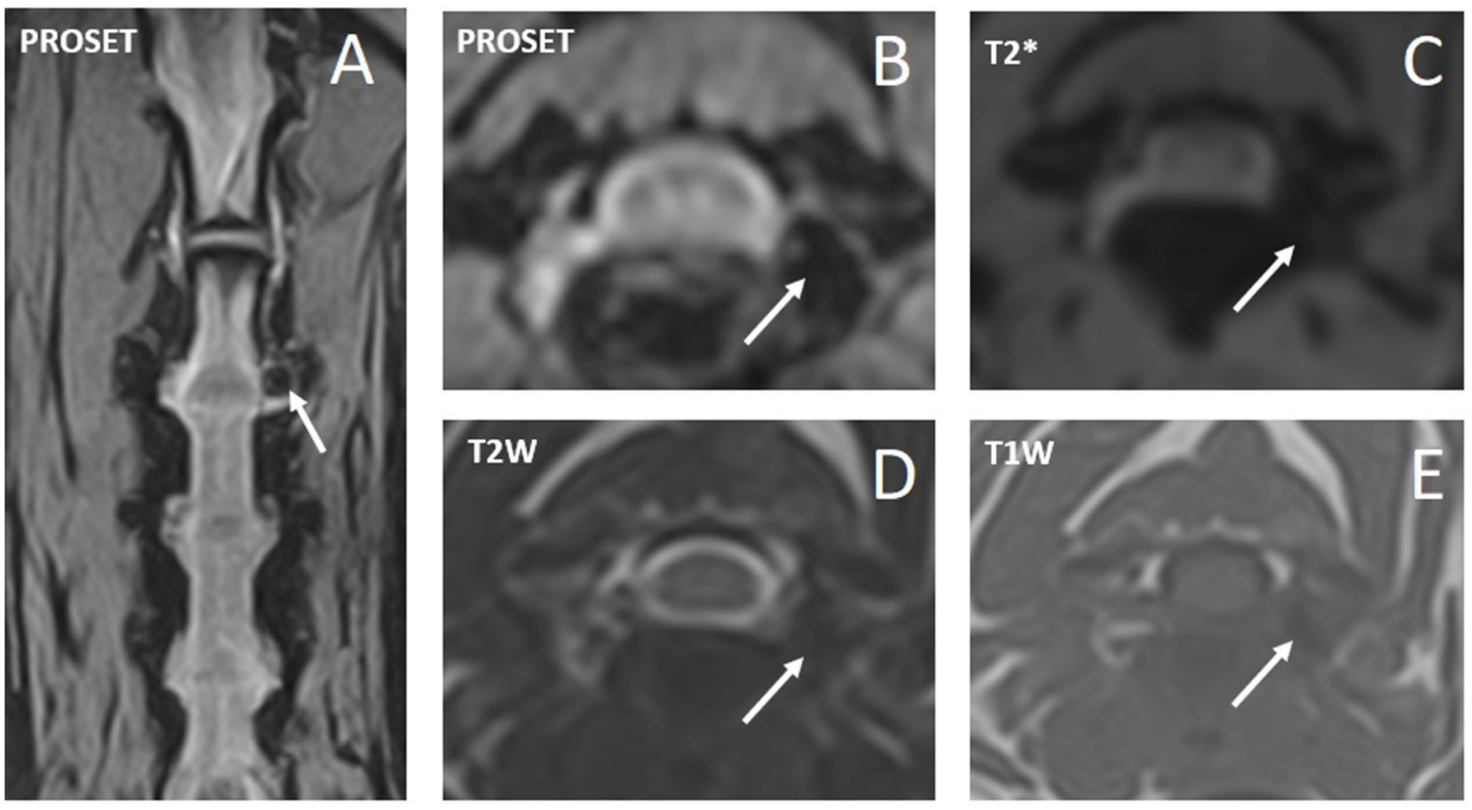
Magnetic resonance images of case #7 with a C3-4 left-sided foraminal and far-lateral intervertebral disk extrusion. A: 3D dorsal nerve root/PROSET (water excitation) sequence – dorsal plane at the level of the C3-4 foramina, B: 3D dorsal nerve root/PROSET (water excitation) sequence – reconstructed transverse plane at the level of the C3-4 intervertebral disk, C: T2*-weighted sequence – transverse plane at the level of C3-4 intervertebral disk, D: T2-weighted sequence – transverse plane as C, E: T1-weighted sequence – transverse plane as C. The white arrows point to the extruded disk material in the foramen and far-lateral position at the level of C3-4 that contrasts with the normal, contralateral side for reference.

**Figure 3:**
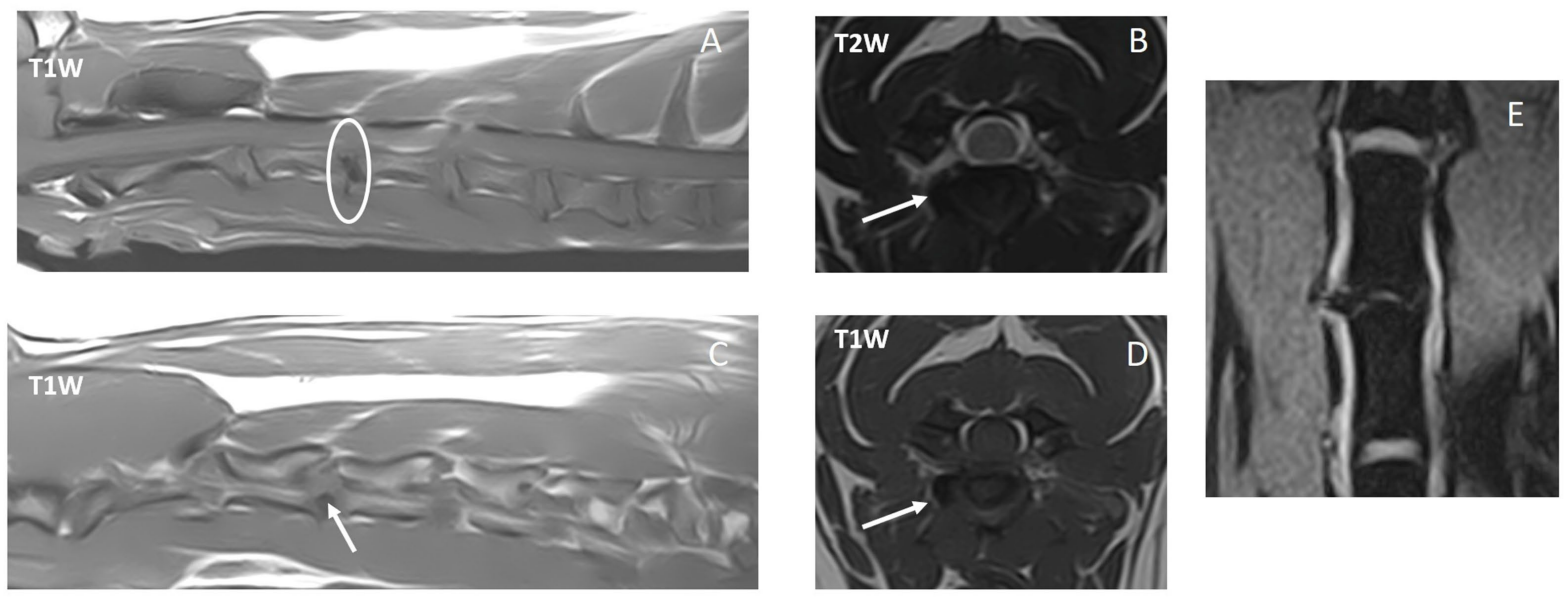
Magnetic resonance images of case #8 with a C3-4 right-sided far-lateral intervertebral disk extrusion. A: T1-weighted sequence – sagittal midplane, B: T1-weighted sequence – sagittal paramedian right-sided at the level of the right pedicle of C4, C: T2-weighted sequence – transverse plane at the level of C3-4 intervertebral disk, D: T1-weighted sequence – transverse plane as C, E: 3D dorsal nerve root / PROSET (water excitation) sequence – dorsal plane at the level of the vertebral arteries. The white circle in A depicts the intervertebral disk space that is hypointense on T1W sagittal images, indicating mineralization. The white arrows point to the T1W and T2W hypointense far-lateral extruded disk material. The vertebral artery on the left in E is interrupted on the right side by far-laterally extruded material.

**Figure 4:**
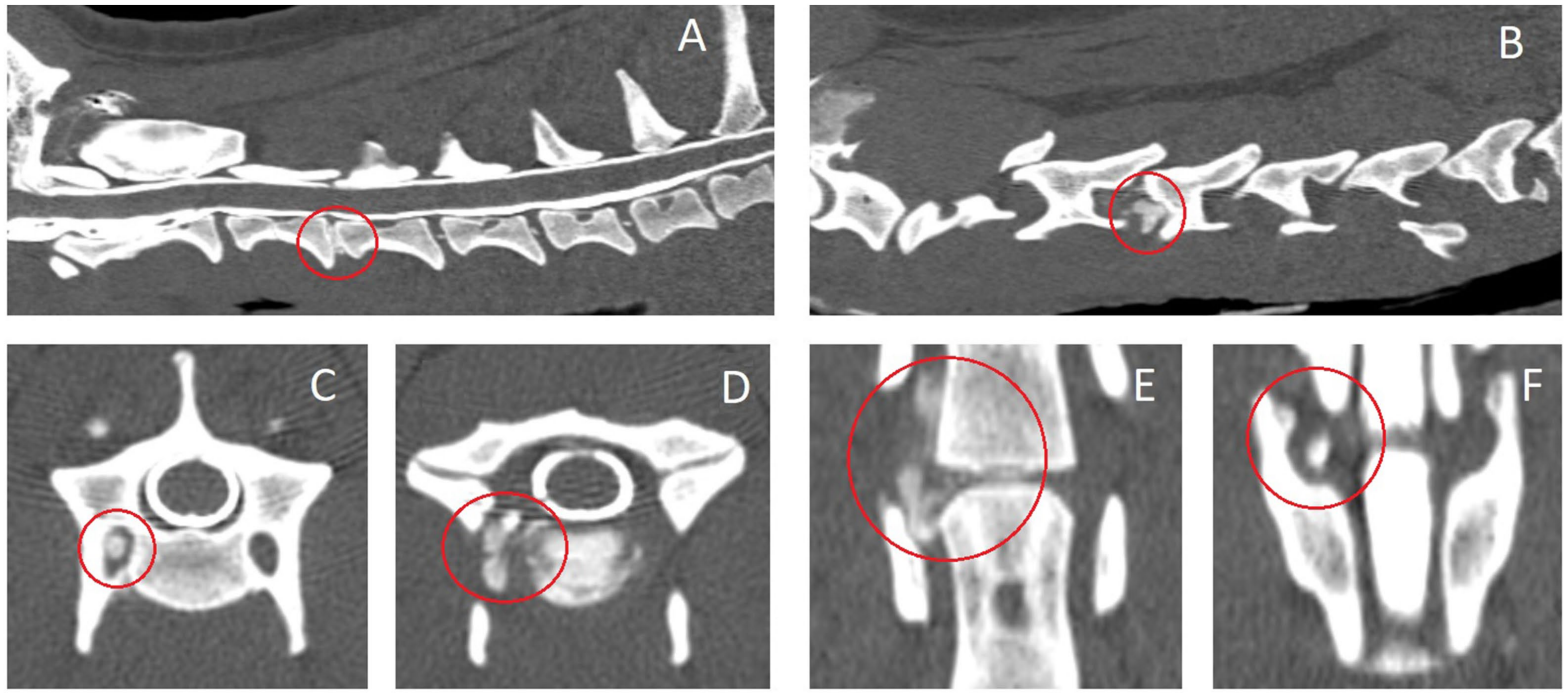
Computed tomographic images (with intrathecal contrast medium – myelogram) of case #2 with a C3-4 right-sided far-lateral and foraminal intervertebral disk extrusion. A: sagittal midplane reconstruction, B: right-sided parasagittal reconstruction at the level of the right pedicle of C4, C: transverse plane at the level of the cranial aspect of C4, D: transverse plane at the level of the C3-4 intervertebral disk, E: dorsal plane reconstruction at the level of the transverse foramen of the C4 vertebra, F: dorsal plane reconstruction at the level of the intervertebral foramen. The affected intervertebral disk and hyperattenuating extruded disk material is circled red.

Table 2 includes a summary of employed diagnostic imaging modalities and findings. Far-lateral cervical IVDEs were diagnosed with high-field MRI in 6/10 (60%) dogs (examples in Figures 2, 3), CT-myelogram in 2/10 (20%) dogs (example in Figure 4), and both CT and low-field MRI in 2/10 (20%) dogs. One dog (1/10, 10%) had both a far-lateral IVDE (C2-3, left) and a far-lateral/foraminal IVDE (C3-4, left). Therefore, we report the features for 11 IVDEs. Far-lateral IVDE was diagnosed in all dogs (10/10, 100%); 6/11 (55%) were combined foraminal/far-lateral IVDEs. Lateralization was to the left in 7/11 (64%) and to the right in 4/11 (36%) IVDEs. All far-lateral and combination of far-lateral/foraminal extrusions were at the level of a narrowed IVD space in comparison to the adjacent IVD spaces, and partially mineralized IVD (hyperattenuating in CT, markedly hypointense in all sequences in MRI). Affected IVD spaces included: C3-4 (6/11, 55%), C5-6 (3/11, 27%), and C2-3 (2/11, 18%). From the CT images, all foraminal and far lateral extrusions were homogeneous and mineralized (hyperattenuating to the spinal cord, HU > 500). From the MRI images, the extradural lesions were slightly heterogeneous but predominantly and markedly hypointense in all sequences, consistent with mineralization. When comparing between MRI sequences, the extradural material was T2W and T1W isointense to the cortical bone, and this resulted in decreased conspicuity in T2W images compared to T1W and T2*W images (Figures 1, 2). In all cases, the extradural lesion caused mass effect and consequent lack of visualization of the vertebral artery and spinal nerve along the length on where the extradural material was sited. From the MRI images, the fat suppression sequences (most of them dorsal STIR) revealed mild ill-defined hyperintensity of the epaxial muscles and perineural tissues around the extruded material. Lateralization of the clinical signs matched with lateralization of the far-lateral IVDE (on imaging) in all cases where lateralization was noted.

**Table 2:** Diagnostic imaging modalities and findings.

	Number (percentage)
Diagnostic imaging modality	
MRI	6/10 (60%)
CT - Myelogram	2/10 (20%)
CT and low-field MRI	2/10 (20%)
Location lateralized IVDE	
Far-lateral + Foraminal	11/11 (100%) 6/11 (55%)
Location (intervertebral disk space)	
C2-3	2/11 (18%)
C3-4	6/11 (55%)
C4-5	0/11 (0%)
C5-6	3/11 (27%)
C6-7	0/10 (0%)
C7-T1	0/10 (0%)
Lateralization	
Left	7/11 (64%)
Right	4/11 (36%)

All cases (10/10, 100%) were managed without surgery and received analgesic medications as primary medical treatment (details including dosages are listed in Table 1). Multimodal analgesic treatment was employed in all cases (10/10, 100%). Medications used included: gabapentinoids (10/10, 100%, gabapentin 7/10 (70%) and pregabalin 3/10 (30%)), non-steroidal anti-inflammatory drugs (NSAIDs (meloxicam or carprofen)) (10/10, 100%), paracetamol (3/10, 30%), methocarbamol (2/10, 20%), tramadol (2/10, 20%), amitriptyline (1/10, 10%), and ketamine (1/10, 10%). Duration of treatment varied, with a median duration of 25 days (range 10–84) for any of the included medications for a particular case. The actual treatment details per case in Table 1 reflect the variability in duration of individual medications and the differences in tapering and non-tapering of different medications in different patients.

Cage rest was recommended in 3/10 (30%) cases (unspecified length or 4 weeks). Short-term outcome (<3 months) was recorded in 9/10 (90%) cases. Resolution of clinical signs was reported in 7/9 (78%) cases. A single case (case #2) was unresponsive to the treatment initiated (carprofen (2 mg/kg q12h for 10 days), pregabalin (4.2 mg/kg q12h for 20 days), tramadol (1 mg/kg q12h for 15 days), paracetamol (9.6 mg/kg q12h for 15 days)). Another single case (case #9) showed persistent lameness as the only remaining clinical sign. Long-term follow-up was available for 6/10 (60%) cases (median 11.5 months, range 5.5–30 months); 5/6 (83%) showed resolution of clinical signs. Recurrence of clinical signs after full resolution was reported in 1 case, 9 months after diagnosis (case #8). This case was managed medically again, without diagnostic imaging, with a successful outcome.

We include the details of 2 particular cases that exemplify some of the remarkable presenting complaints and histories that were recorded.

### Case example: case #7

A 7-year-old female entire 5 kg dachshund was presented with a 2-week history of attacks of abnormal cervical posture and vocalization, which was waning in frequency but still occurred multiple times per day (3–6 times, 10–60 s each). Videos provided by the owner showed fasciculations of cervical muscles and cervical pseudodystonia with ventroflexion and head tilting/turning (torticollis) (Supplementary Video S1). No vocalization was heard on the videos nor reported by the owners. General clinical examination was unremarkable. Neurological examination was unremarkable and most notably no signs of cervical hyperesthesia were elicited upon palpation. The dog was able to shake its head with vigor after instilling a drop of water in the external ear canal (drop-in-ear test). This test is frequently used by one of the authors (KS) to assess the ability to perform a normal, vigorous head shaking movement. Hesitancy, incomplete performance or the occurrence of signs of pain (vocalization) when the dog (attempts to) exhibit this behavior is interpreted as abnormal. High-field MRI revealed a left-sided C3-4 far-lateral and foraminal IVDE (Figure 2). Treatment was initiated with meloxicam (0.1 mg/kg q24h for 2 weeks) and gabapentin (10 mg/kg q8h for 2 weeks, followed by q12h for 1 week and then diskontinued). One recurrent attack was reported in the first 2 days of treatment, thereafter no recurrence was noticed. Total follow-up was 10 months without recurrence of signs.

### Case example: case #8

An 8-year-old female neutered 9 kg crossbreed dog (with a chondrodystrophic phenotype) was presented with a 2-week history of acute onset recurrent episodic cervical hyperesthesia, vocalization (‘pain attacks’ or yelping episodes), and right thoracic limb lameness / nerve root signature. Based on suspected epileptic seizures, the dog had been prescribed phenobarbital 2 mg/kg q12h by the referring veterinarian without resolution of clinical signs. The owner had made some videos of the attacks and period after an attack at home (Supplementary Videos S2 and S3). These showed a subjectively conscious dog, with a guarded posture, nerve root signature, and high-pitched vocalization (yelping) of 30 s to 2 min in duration. After the episode, the dog would be subjectively lethargic, exhibited a nerve root signature of the right thoracic limb, and frequently licked its nose. This was interpreted as post-ictal signs by the referring veterinarian. General clinical examination was unremarkable. Neurological examination showed cervical hyperesthesia mainly when the neck was flexed to the left and deep palpation was performed over the right mid-cervical area dorsal to the trachea and ventral to the neck extensor musculature. After deep palpation, fasciculations were palpable superficially in the lateral right-sided cervical musculature. The dog was hesitant to shake its head and only wobbled its head slightly upon the drop-in-ear test. There was mild general proprioceptive ataxia of the pelvic limbs attributed to the use of phenobarbital. Otherwise, the neurological examination was within normal limits. High-field MRI revealed a right-sided C3-4 far-lateral IVDE (Figure 3). Medical treatment was started with carprofen (2 mg/kg q12h for 2 weeks) and gabapentin (11 mg/kg q8h for 12 weeks). Amitriptyline (1.1 mg/kg q12h) was added due to recurrent episodes of pain after a week. This treatment was effective and tapered slowly over the next 2 months (first carprofen, then gabapentin, then amitriptyline). A recurrence of pain signs was noticed 9 months after first presentation (in remission for 7 months prior) and managed with meloxicam and gabapentin only for 2 and 4 weeks, respectively. No further diagnostic tests were performed at that time. No signs of pain were reported thereafter, with a long-term follow-up of 18 months after first presentation.

## Discussion

In this retrospective multicenter study, we report the clinical features, diagnosis, treatment, and outcome of cervical far-lateral and combined cervical far-lateral/foraminal IVDE in 10 dogs. A previous publication, in which diagnosis was based on radiography, myelography, and surgical findings, describes 7 dogs that were presented for neck pain and/or nerve root signature (14). Of those cases, 2/7 were diagnosed with ‘lateral’ intervertebral disk extrusions (a 6-year-old Poodle and a 10-year-old Dachshund) (14). The term ‘lateral’ in that report refers to what we call ‘far-lateral’, in accordance with recent literature (12). The myelographic studies contributed mainly to exclude compression on the dural tube, while the oblique radiographs helped to confirm the presence of opaque material in the intervertebral foramen (“fogging” of the foramen) (14). Surgery consisted of hemilaminectomy with facetectomy in all cases (3 cases had a ventral slot technique performed in 5–15 days before second surgery, during which no material was retrieved). The surgery confirmed a foraminal position of the extruded material in 5/7 cases and a (far-) lateral position in 2 cases. All cases also received various medical treatments.

In our study, 10 dogs had 11 IVDEs: 6/11 were combined far-lateral/foraminal IVDEs and 5/11 were pure far-lateral IVDE (i.e., extruded material was located distal to the foramen, lateral to the IVD). Far-lateral IVDEs in dogs are reported most frequently in the thoracolumbar area, mostly affecting the caudal lumbar IVDs (L5-6 and L6-7) (7). The lack of publications pertaining to cervical far-lateral IVDEs suggest that it is rarely diagnosed. Awareness of this type of IVDE is still of vital importance to clinicians, especially those performing and interpreting diagnostic imaging studies. All foraminal/far-distal disk extrusions included in this study were mineralized and were at the level of a narrowed intervertebral disk space with a partially mineralized intervertebral disk. As mineralized lesions and bone structures are well evaluated from CT images, these lesions were obvious and easily diagnosed by CT. Mineralization may be more challenging to depict from MRI studies, and therefore, when suspecting a foraminal/far-distal disk extrusion, it is important to include several sequences and several planes.

Based on these considerations and the findings in our study, we recommend the following to optimize the chance of correct diagnosis of far-lateral cervical IVDE on MRI studies:

- Include sagittal T2W and sagittal T1W in order to visualized a narrowed intervertebral mineralized disk (hypointense in T2W and hypointense in T1W).
- Dorsal fat suppression sequences (such as STIR, DIXON), in order to assess any lateralized abnormal signal intensity of paravertebral and perineural tissues.
- Transverse T2W, T1W and T2* at the level of the suspected foramina/far-distal extrusion from the level of the mid-body of cranial vertebra to the mid-body of caudal vertebra in respect to the IVD space of interest. An IVD of interest may be defined as any IVD showing signal characteristics consistent with degeneration (e.g., T2-hypointensity of the nucleus pulposus).The combination of these three sequences will help to visualize accurately the extruded disk material and corroborate mineralization (hypointense in all sequences, better depicted in T2*). These transverse planes will allow the assessment of the spinal nerves and vertebral arteries.
- Select appropriate slice thickness, ideally 2.5 mm when using the sequences above.
- PROSET (water excitation) images may be added to assess further the spinal nerves, and assess their size and course.

Regarding CT, parasagittal reconstructed CT images show the presence of hyperattenuating material laterally if the disk material is calcified (degenerated) (Figure 4). The use of non-ionic intrathecal contrast medium (CT-myelogram or myelo-CT) in this particular type of IVD disease might not add any value unless there is concomitant spinal cord compression by part of the extruded disk material or additional spinal cord compressions affecting other IVD spaces. When intravenous non-ionic contrast medium is used, however, a loss of continuity of the signal of the vertebral artery might be revealed more conspicuously as with MRI studies. Like on MRI, dorsal plane reconstruction and transverse plane images at the level of the IVD can provide further confirmation and evaluation of lateralized extruded disk material. The distribution of mineralized extruded disk material may be even more conspicuously visualized on CT images than on MRI images. This is exemplified in Figure 4C where the extruded and calcified disk material can be visualized in the cranial aspect of the transverse foramen, compressing the structures therein (e.g., vertebral artery).

While we do not report the use of radiography and myelography (other than CT-myelography), Felts and Prata (1983) have demonstrated the usefulness of oblique radiographs specifically (14). However, cross-sectional imaging offers considerable advantages (e.g., ease of obtaining diagnostic images and improved ability to localize the IVDE).

The clinical sign of cervical hyperesthesia is exhibited by most dogs affected by far-lateral as well as foraminal IVDEs. In a recent study on canine thoracolumbar far-lateral or foraminal IVDEs, 92% (35/38) exhibited signs of pain (7). All dogs had either pain or pelvic limb lameness. Two dogs did not show signs of pain on examination, but intermittent yelping episodes were reported by the owner. These episodes mirror the ‘pain attacks’ reported by the owners in the cases reported in our study. In a case series on cervical foraminal IVDE, all 13 dogs included exhibited signs of pain (12). Nerve root signature or lameness was reported in most dogs in both publications (7, 12). Nerve root signature or lameness was reported 5/10 cases in our study. Nerve root signature is more often reported when spinal nerves that contribute to either the brachial or lumbosacral plexus are involved. More than half of the cases reported in our study affected the C3-4 IVD, while in a recent study on nerve root signature in dogs with cervical IVDE the most commonly affected IVD space was C6-7 (15). Interestingly, the report from 1983 mentions that nerve root signature is a common feature for extrusions affecting nerve roots (or actually usually spinal nerves) from C4 caudally, and does not occur for lesions affecting those of C1-3 (14). The C4 spinal nerve exits at C3-4. Our study included dogs with cervical IVDEs at C3-4 with nerve root signature as a clinical sign. The reason for nerve root signature due to lesions cranial to the segments and associated nerve roots contributing to the brachial plexus is debated and remains speculative (14).

The ‘pain attacks’ reported by owners and documented on video have the potential of being misinterpreted as epileptic seizures. This is evidenced by the fact that case #9 was prescribed phenobarbital by the referring veterinarian based on a suspicion of epileptic seizure activity. There are several mimics of epileptic seizures (16), including syncope, narcolepsy/cataplexy, neuromuscular weakness, paroxysmal behavior changes, vestibular attacks, paroxysmal dyskinesia and idiopathic head tremor (17). Episodic pain has to be considered when owners report ‘seizures’ or ‘fits’. The lack of phenobarbital responsiveness called into question an epileptic nature of the seizures reported by the owner in case #9. Careful examination of video footage and in depth questioning of the owner for tell-tale signs of epileptic seizure characteristics (e.g., post-ictal signs and autonomic signs) may help in the differentiation between all these differential diagnoses.

Irritation or injury to spinal nerves can result in the clinical phenomenon of ‘peripheral nerve hyperexcitability’, leading to electrical discharges in nerves and seemingly spontaneous muscle contractions termed ‘fasciculations’ (18). A lateralized extruded disk will compress soft tissues adjacent to it, including spinal nerves and branches thereof as they course toward their intended targets. Fasciculations may be felt (palpated) or seen as flickers of muscle fibers contracting underneath the skin. Although these fasciculations are not voluntarily elicited (i.e., they are involuntary), they cannot be regarded as truly spontaneous since there is an identifiable cause for their occurrence in the case of nerve fiber compression. Alternatively, such muscle activity in the cervical region has been called ‘cervical jerks’ in recent literature (19).

Case #7 displayed a remarkable clinical sign that we, based on human literature, have termed (episodic) cervical pseudodystonia. Dystonia is referred to in literature variably as a clinical sign (“dys-” = bad, ill, or abnormal, and “-tonia” from “tonos” = tension or tone (muscle-)) or a disorder in and of itself (20). A consensus report from human literature defines dystonia as *“a movement disorder characterized by sustained or intermittent muscle contractions causing abnormal, often repetitive, movements, postures, or both”* (21). Cervical dystonia, as one clinical form of dystonia in particular, is fairly common in humans and can be continuous or episodic (21–23). However, as cervical dystonia refers specifically to a (mostly idiopathic, syndromic, and/or genetic) movement disorder, all other causes of abnormal cervical posturing that look like cervical dystonia are termed ‘pseudodystonia’ (24, 25). Non-exhaustive lists in human literature mention many possible causes of (cervical) pseudodystonia (24, 25). Possible causes entail lesions or disorders affecting the central nervous system, peripheral nervous system, as well as non-neurological disorders. In cases of far-lateral IVDE, the peripheral nervous system is affected. The dog in case #7 was diagnosed with a C3-4 left-sided foraminal and far-lateral IVDE, and the abnormal posturing was relieved by treatment with meloxicam and gabapentin, and did not recur after discontinuation of treatment.

Regarding treatment options, both surgical and medical treatment were associated with good to excellent outcomes in 90% or more in dogs with thoracolumbar foraminal or far-lateral IVDEs (7). Most dogs with cervical foraminal IVDE were reported to recover completely with only medical therapy in one study (12). Likewise, most of our cases responded favorably to medical treatment. In case #9 reported here, medical therapy with only carprofen and gabapentin failed in the short term and the addition of amitriptyline resulted in amelioration of ‘pain attacks’. Amitriptyline is a tricyclic antidepressant used for the treatment of neuropathic pain in people and also utilized in various conditions thought or proven to be associated with neuropathic pain in animals (26–28). Its use for the treatment of pain related to IVDE in dogs has not been evaluated to date. The positive effect of the addition of amitriptyline in one case reported here suggests that it could be considered as a treatment addition when other medical treatment strategies fail. Perineural injections have been reported as a treatment option for lateralized cervical IVDEs (29). In that report, all cases had a foraminal IVDE, but based on the images in the report, there was at least one case with a combined foraminal/far-lateral extrusion. This treatment option could be considered in the treatment of cervical far-lateral IVDE, though it was not employed in cases included in our study.

Surgical treatment was not employed in any case reported here. Although specific reasons were not noted, expected difficulties with a lateral approach to material contacting the vertebral artery likely contributed to a preference for medical treatment. A dorsal approach was reported to be successful for both foraminal and far-lateral IVDE (14). Still, the removal of lamina, pedicles and facet joints in the technique reported and depicted in the article are not ideally suited to reach a far-lateral IVDE. The usefulness of physiotherapy, (crate or bench) rest, or other types of complementary treatment modalities or recommendations (e.g., laser therapy and acupuncture) in canine cases of far-lateral IVDE has not been evaluated. These may still be considered, despite lack of specific efficacy.

There are several limitations to this study. Since this was a multicenter, retrospective study, diagnostic procedures, treatment, and follow-up were not standardized. With regard to follow-up, this also meant that we were unable to specifically assess the time to adequate effect of medications or resolution of clinical signs. While one might expect that owners will soon reach out to report unsatisfactory effect of analgesic medications, the authors’ experiences vary with respect to owners reporting adequacy of the effect of treatment. Some may only report such when prompted at follow-up (live or via telephone). For example, the owner may report an improvement when prompted, but an unsatisfactory one, leading to a decision to add medications to the treatment. Since multimodal analgesic treatment was employed in all patients, we cannot assess which medications most benefitted the patients specifically. Neither can we assess which combination would be specifically recommended to treat patients with cervical far-lateral IVDE. Nonetheless, in general, the adage ‘whatever works’ would apply with respect to analgesic medications for the treatment of such patients (i.e., striving for adequate analgesia is paramount). The plethora of different combinations, dosages, and tapering schemes employed for the patients in our study may be seen as a reflection of this consideration.

Another limitation is the fact that there were no standardized imaging protocols for patients presented for the clinical signs as reported in this study that were specifically designed to increase the chance of diagnosis of cervical far-lateral IVDEs. Additionally, a lack of mineralization in extruded disk material may contribute to missing a diagnosis of far-lateral IVDE on CT images, and not including all IVD spaces or dorsal imaging planes may contribute to missing the diagnosis on MRI studies. Therefore, we cannot determine or speculate on the prevalence or possible underdiagnosis of cervical far-lateral disk extrusions in dogs.

Finally, we report recurrence of clinical signs in only one case. However, as no diagnostic procedures were performed at the time of recurrence of signs, it may well be that a different disorders or a different location accounted for the clinical signs at the time of recurrence. In other words, recurrence of clinical signs does not equal recurrence of clinical signs due to the same cervical far-lateral or foraminal and far-lateral IVDE.

In conclusion, cervical far-lateral disk extrusions are a rare clinical entity in dogs, but can result in severe, possibly episodic, pain. Medical management is associated with a favorable short- and long-term outcome in most cases.

## Data Availability

The original contributions presented in the study are included in the article/Supplementary material, further inquiries can be directed to the corresponding author.

## Supplementary material

Supplementary Video S1 Case #7 – episodic signs, cervical pseudodystonia.

Supplementary Video S2 Case #8 – ‘pain attack’.

Supplementary Video S3 Case #8 – post-attack signs.

## References

[1] Da Costa RC De Decker S Lewis MJ Volk H. Canine spinal cord injury consortium (CANSORT-SCI). Diagnostic imaging in intervertebral disc disease. Front Vet Sci. (2020) 7:588338. doi: 10.3389/fvets.2020.588338, PMID: 33195623 PMC7642913

[2] Fenn J Olby NJ. Canine spinal cord injury consortium (CANSORT-SCI). Classification of intervertebral disc disease. Front Vet Sci. (2020) 7:579025. doi: 10.3389/fvets.2020.579025, PMID: 33134360 PMC7572860

[3] Moore SA Tipold A Olby NJ Stein V Granger N. Canine spinal cord injury consortium (CANSORT SCI). Current approaches to the Management of Acute Thoracolumbar Disc Extrusion in dogs. Front Vet Sci. (2020) 7:610. doi: 10.3389/fvets.2020.00610, PMID: 33117847 PMC7521156

[4] Spitzbarth I Moore SA Stein VM Levine JM Kühl B Gerhauser I. Canine spinal cord injury consortium (CANSORT-SCI). Current insights into the pathology of canine intervertebral disc extrusion-induced spinal cord injury. Front Vet Sci. (2020) 7:595796. doi: 10.3389/fvets.2020.595796, PMID: 33195632 PMC7653192

[5] Bergknut N Egenvall A Hagman R Gustås P Hazewinkel HA Meij BP. Incidence of intervertebral disk degeneration-related diseases and associated mortality rates in dogs. J Am Vet Med Assoc. (2012) 240:1300–9. doi: 10.2460/javma.240.11.1300, PMID: 22607596

[6] Casado D Fernandes R Lourinho F Gonçalves R Clark R Violini F. Magnetic resonance imaging features of canine intradural/extramedullary intervertebral disc extrusion in seven cases. Front Vet Sci. (2022) 9:1003042. doi: 10.3389/fvets.2022.1003042, PMID: 36187811 PMC9517942

[7] Silva S Guevar J José-López R De Decker S Brocal J de la Fuente C. Clinical signs, MRI findings and long-term outcomes of foraminal and far lateral thoracolumbar intervertebral disc herniations in dogs. Vet Rec. (2022) 190:e1529. doi: 10.1002/vetr.1529. Epub 2022 Mar 12, PMID: 35278224

[8] Bagley RS Pluhar GE Alexander JE. Lateral intervertebral disk extrusion causing lameness in a dog. J Am Vet Med Assoc. (1994) 205:181–5. doi: 10.2460/javma.1994.205.02.181 7928570

[9] Bagley R Tucker R Harrington M. Lateral and foraminal disk extrusion in dogs. Comp Cont Educ Pract Vet. (1996) 18:795–805.

[10] Fadda A Lang J Forterre F. Far lateral lumbar disc extrusion: MRI findings and surgical treatment. Vet Comp Orthop Traumatol. (2013) 26:318–22. doi: 10.3415/VCOT-12-08-0106, PMID: 23857574

[11] Kim J Kim H Hwang J Eom K. Far lateral lumbar disc extrusion in a dachshund dog. Korean J Vet Res. (2019) 59:165–9. doi: 10.14405/kjvr.2019.59.3.165

[12] Bersan E McConnell F Trevail R Behr S De Decker S Volk HA. Cervical intervertebral foraminal disc extrusion in dogs: clinical presentation, MRI characteristics and outcome after medical management. Vet Rec. (2015) 176:597. doi: 10.1136/vr.102851, PMID: 25745084

[13] Gödde T Steffen F. Surgical treatment of lumbosacral foraminal stenosis using a lateral approach in twenty dogs with degenerative lumbosacral stenosis. Vet Surg. (2007) 36:705–13. doi: 10.1111/j.1532-950X.2007.00324.x, PMID: 17894598

[14] Felts JF Prata RG. Cervical disk disease in the dog—intraforaminal and lateral extrusions. J Am Anim Hosp Assoc. (1983) 19:755–60.

[15] Schachar J Bocage A Nelson NC Early PJ Mariani CL Olby NJ. Clinical and imaging findings in dogs with nerve root signature associated with cervical intervertebral disc herniation. J Vet Intern Med. (2024) 38:1111–9. doi: 10.1111/jvim.16982, PMID: 38216520 PMC10937489

[16] Berendt M Farquhar RG Mandigers PJ Pakozdy A Bhatti SF De Risio L. International veterinary epilepsy task force consensus report on epilepsy definition, classification and terminology in companion animals. BMC Vet Res. (2015) 11:182. doi: 10.1186/s12917-015-0461-2, PMID: 26316133 PMC4552272

[17] De Risio L Bhatti S Muñana K Penderis J Stein V Tipold A. International veterinary epilepsy task force consensus proposal: diagnostic approach to epilepsy in dogs. BMC Vet Res. (2015) 11:148. doi: 10.1186/s12917-015-0462-1, PMID: 26316175 PMC4552251

[18] Cerda-Gonzalez S Packer RA Garosi L Lowrie M Mandigers PJJ O'Brien DP. International veterinary canine dyskinesia task force ECVN consensus statement: terminology and classification. J Vet Intern Med. (2021) 35:1218–30. doi: 10.1111/jvim.16108 33769611 PMC8162615

[19] Olender M Couturier J Gatel L Cauvin E. Cervical jerks as a sign of cervical pain or myelopathy in dogs. J Am Vet Med Assoc. (2023) 261:510–6. doi: 10.2460/javma.22.11.0507 36735506

[20] Santifort KM Mandigers PJJ. Dystonia in veterinary neurology. J Vet Intern Med. (2022) 36:1872–81. doi: 10.1111/jvim.16532, PMID: 36086931 PMC9708431

[21] Albanese A Bhatia K Bressman SB Delong MR Fahn S Fung VS. Phenomenology and classification of dystonia: a consensus update. Mov Disord. (2013) 28:863–73. doi: 10.1002/mds.25475, PMID: 23649720 PMC3729880

[22] di Biase L Di Santo A Caminiti ML Pecoraro PM Di Lazzaro V. Classification of dystonia. Life (Basel). (2022) 12:206. doi: 10.3390/life12020206, PMID: 35207493 PMC8875209

[23] Velickovic M Benabou R Brin MF. Cervical dystonia: pathophysiology and treatment options. Drugs. (2001) 61:1921–43. doi: 10.2165/00003495-200161130-00004 11708764

[24] Berlot R Bhatia KP Kojović M. Pseudodystonia: a new perspective on an old phenomenon. Parkinsonism Relat Disord. (2019) 62:44–50. doi: 10.1016/j.parkreldis.2019.02.008, PMID: 30819557

[25] Raju S Ravi A Prashanth LK. Cervical dystonia mimics: a case series and review of the literature. Tremor Other Hyperkinet Mov (N Y). (2019) 9:707. doi: 10.7916/tohm.v0.707 PMC6898896 31867135

[26] Grubb T. Chronic neuropathic pain in veterinary patients. Top Companion Anim Med. (2010) 25:45–52. doi: 10.1053/j.tcam.2009.10.007 20188338

[27] Mathews KA. Neuropathic pain in dogs and cats: if only they could tell us if they hurt. Vet Clin North Am Small Anim Pract. (2008) 38:1365–414, vii-viii. doi: 10.1016/j.cvsm.2008.09.001, PMID: 18954689

[28] Moore SA. Managing neuropathic pain in dogs. Front Vet Sci. (2016) 3:12. doi: 10.3389/fvets.2016.00012, PMID: 26942185 PMC4762016

[29] Giambuzzi S Pancotto T Ruth J. Perineural injection for treatment of root-signature signs associated with lateralized disk material in five dogs (2009-2013). Front Vet Sci. (2016) 3:1. doi: 10.3389/fvets.2016.00001, PMID: 26858952 PMC4728328

