# Erratum: Case series: cervical far-lateral and combined cervical far lateral/foraminal intervertebral disk extrusions in 10 dogs

**DOI:** 10.3389/fvets.2025.1609269

**Published:** 2025-04-17

**Authors:** Frontiers Production Office

**Affiliations:** Frontiers Media SA, Lausanne, Switzerland

**Keywords:** herniation, hyperesthesia, chondrodystrophic, pain, nerve root signature

Due to a production error, Dr. Ruggeri, Dr. Alcoverro and Dr. Tauro's affiliations were assigned incorrectly. The correct affiliations are:

Marco Ruggeri^4,5^

^4^ChesterGates Veterinary Specialists, Chester, United Kingdom

^5^Department of Clinical Sciences, North Carolina State University, Raleigh, NC, United States

Emili Alcoverro^4,6^

^4^ChesterGates Veterinary Specialists, Chester, United Kingdom

^6^AniCura Ars Veterinària Hospital Veterinari, Barcelona, Spain

Anna Tauro^4,5^

^4^ChesterGates Veterinary Specialists, Chester, United Kingdom

^5^Department of Clinical Sciences, North Carolina State University, Raleigh, NC, United States

The publisher apologizes for this mistake. The original article has been updated.

